# Complexity in Global Health– Bridging Theory and Practice

**DOI:** 10.5334/aogh.3758

**Published:** 2022-07-01

**Authors:** Carlos A. Faerron Guzmán

**Affiliations:** 1 The Graduate School, University of Maryland, Baltimore, 620 W Lexington St, Baltimore, MD 21201, USA

**Keywords:** Complex systems, globalization, systems thinking, transdisciplinarity, interconnection

## Abstract

Increasingly, health reflects an integrated outcome of a growing globalized system. Economic, political, cultural, environmental, and other global processes profoundly influence how we understand and approach health challenges. As these occur in a webbed, dynamic, and interdependent fashion, health can be viewed as a complex issue. Drawing from this understanding, in this viewpoint, I assert applying complexity theory to produce a definition of the field of global health. Complexity theory tenets such as non-linearity, transdisciplinarity, open-system analysis, and global-local phenomenology can provide a theoretical basis for a substantive understanding of global health phenomena and a richer instrumental approach to global health challenges. Harmonization between complexity theory and global health may provide the foundation to close the health equity gap put forth by the global health agenda.

## Introduction

Today, global health is a diverse field of research, practice, and education whose boundaries are notoriously difficult to determine. It is taught in various formats, including undergraduate education, master’s degrees, and doctoral programs [1,2]. It is practiced in multiple contexts, such as resource-constrained communities and highly specialized clinical facilities. Research topics in the field also vary widely, spanning from vaccine development to understanding the role of inequities in health outcomes. Nonetheless, and partly due to the open interpretation and understanding of the evolving global health field, there remains continuous debate on what global health actually is and who it is for [3].

**Figure 1:**
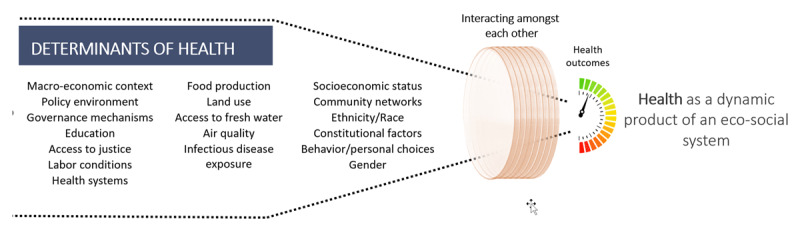
Health as an outcome of a global/local eco-social system.

In this ongoing debate, it is central to understand the broader meaning of *health* in global health. Health is an outcome of social, cultural, political, economic, and environmental processes [4,5,6]. Concomitantly, and as has been demonstrated by the COVID-19 pandemic, the current global context is exacerbated by the accelerated process of globalization and its influence on health outcomes [7]. As a result, health can be regarded as an integrated outcome of a growing globalized system. None of these processes or systems exist in a vacuum; on the contrary, they occur in a webbed, dynamic, and interdependent fashion, i.e., complex (Figure 1) [8,9,10].

Additionally, and just as relevant to this conceptual debate, there is a current emphasis on shifting global health’s instrumental and practical focus. Authors like Frenk et al [10]. and Myers and Frumkin [11] assert that global/planetary health practice must focus on understanding the multidimensionality of health, the continually evolving systems that underpin it, and the interlinkages between these systems. It is argued that once this is achieved, global health practitioners will be able to work across sectors to enact a wide variety of comprehensive solutions to global health challenges [12].

## Current Gaps in Global Health Theory and Practice

Global health has its historical roots in tropical medicine and international health, a dominantly colonial and neo-colonial perspective of medicine and public health, respectively, and has primarily borrowed conceptual frameworks from these two fields [13]. Consequently, in the early stages of the global health surge, global health practitioners had a predominantly biomedical focus applying vertical and uni-disciplinary approaches that mostly lacked contextual understanding [14,15]. For example, during the 1990’s global health mainly focused on vertical programs battling tuberculosis, HIV, and malaria in lower and middle-income countries [16]. Despite the increased funding streams towards these types of programs, the results fell short of expectations [17].

These shortcomings, accompanied by the resurgence of interest in the Lalonde Report of 1974 [18] and the 1978 Declaration of Alma Ata [19], in parallel with the upsurge of the Millennium Development Goals agenda and the merging of global health with development practitoners, led to the incorporation of a broader community of practice in global health [20]. This paradigmatic shift meant that global health would incorporate and apply various conceptual and theoretical frameworks into its work. These included social theory, applied economics, development theory, human rights, and ethics.

Despite the integration of these fields within global health, global health today is still dominated by international clinical practice and biomedical approaches [21]. The latter is evidenced by the overwhelming majority of global health practitioners trained as physicians or public health specialists, and by the fact that most global health programming is created from medical and public health institutions, which influences the channels of scientinfic distribution [21,22].

Within this context, and despite the call for integration from stakeholders throughout the global health arena, the field is at a discursive impasse dominated by debates on the object, types, and purpose of knowledge in global health [15]. For example, global health as the study of globalization and health; global health as the study of transnational health issues; global health as the way towards global equity in health; global health as international public health [20,23,24]. This impasse has partly been due to gaps in the theoretical frameworks that can be uniformly applied to global health, especially one that could capture the dynamism and multilayered realities of global health today [25].

Furthering the divide, existing global health frameworks pose a synthetic view of global health processes, isolating them in geo-spatial silos and into different fields of practice. For example, within the field of global health, anthropologists studying the relationships of culture and health, economists understanding the impact of international trade on health, clinicians studying the pathophysiology of neglected diseases, and human rights specialists understanding the implications of international treaties in health policy, to name a few. Orphaned of a comprehensive theory to guide global health practice, global health will continue to be, as Paul Farmer stated almost ten years ago, merely a “collection of problems” [25].

The implications for this lack of clarity in definition and theory for global health go beyond pure semantics. Without an established agreement on what we intend to mean by global health, as Koplan et al. state, “we cannot possibly reach agreement about what we are trying to achieve, the approaches we must take, the skills that are needed and the ways that we should use resources” [23]. Furthermore, this deficiency in global health theory, as argued by Arthur Kleinman, “…may or may not have slowed progress in developing and implementing programs, but it surely has limited the education of practitioners and the emergence of an intellectually robust field” [26].

Acknowledging this disconnect of global health from theoretical underpinnings, Kleinman asserts the importance of approaching global health from a theoretical perspective. In his arguments, and as one of the few scholars that have attempted to approach global health from a theoretical lens, Kleinman [26] advocates for social theory in global health practice. Although he argues for a variety of perspectives being brought into global health theory and practice (i.e., structural violence, the social construction of reality, biopower, and purposive action), he concludes that his approach is far from exhaustive and calls for further discussion for the generalization of practice and the construction of knowledge in global health [26].

Most recently, there has been a shift in how scholars and other actors are starting to address global health. For example, Arya and Allison [12] explicitly address the complexity of global health phenomena, making a call to change global health practice and making it “less about service delivery and more about understanding and addressing the deeper structural issues” [12]. The intention is also to capture the multifaceted aspects of global health practice while highlighting that global health actors must come together and create local and global networks that span sectors and scientific disciplines.

Similarly, efforts to frame global health education in an interconnected and interdependent world have also sprung forward. Both the Association of Schools and Programs of Public Health and the Consortium of Universities of Global Health have made significant contributions to creating competency domains in global health that expand beyond international clinical practice (e.g., ethical reasoning, program management, capacity strengthening, strategic analysis) [1,27]. Concomitantly, the discourse has also moved towards the need for interprofessional education in a collaborative environment. Rowthorn and Olsen [28] emphasize that given the complex realities of global health practice, interprofessional education and subsequent work are vital to meet global health demands.

Nonetheless, despite these *new* commonalities within current global health discourse and the recognition of these interwoven realities, emphasized even more during the COVID-19 syndemic, the global health community has not matched this shift with an equal response to create a shared framework of both knowledge and instrumental approaches [29]. Hyper-specialization, power/knowledge imbalances among and across disciplines, irreconcilable epistemological differences, reductionism in theory and practice, institutional structures, and administrative hurdles might be behind these gaps [8,10]. This lack of theory used to inform and construct global health knowledge/practice, and furthermore, this diverse understanding of what is meant by global health, has led, in many cases, to an unstructured, inefficient, and disorganized response to the pressing health issues of today.

## Towards Complexity

Complexity science is a discipline that has been on the rise for at least 50 years. Most recently, there has been a resurgence of complexity theory (CT) in the scientific community, partly explained by the increasing impacts of globalization and the push for interdisciplinary work in the face of complex challenges. Complexity theory and science are, in essence, the study of complex adaptive systems (CAS) [30]. In contrast with traditional Newtonian and Cartesian sciences, CT avoids reductionist approaches that break down phenomena and systems into simple and smaller components. As Morin [31] states:

In opposition to reduction, complexity requires that one tries to comprehend the relations between the whole and the parts. The knowledge of the parts is not enough, the knowledge of the whole as a whole is not enough … Thus, the principle of reduction is substituted by a principle that conceives the relation of whole-part mutual implication. The principle of disjunction, of separation (between objects, between disciplines, between notions, between subject and object of knowledge), should be substituted by a principle that maintains the distinction, but that tries to establish the relation.

CT focuses on the dynamics of interdependence between system components and the properties emerging from these interactions. It also studies non-linear relationships, uncertainty, feedback mechanisms, and adaptation amongst systems. CT considers context and historical trajectories to understand systems’ behavior [30,32]. Furthermore, complexity theory emphasizes “methodological pluralism” [33] as a central mechanism for understanding diverse, multidimensional phenomena. Such CT concepts have been applied to systems such as the human brain, stock markets, infectious diseases, and ecosystems [34]. A key difference from complementary approaches, like systems thinking and socio-ecological models, is the unique pathways that emerge from complex analysis as different ways of knowing interact amongst each other [30,32].

In the last half-century, complexity theory has influenced various fields, including climate research, economics, and biology. In the area of health, CT has been applied to clinical medicine [35], nursing [33], health services management [36], and health policy [37], among others. However, complexity theory has seldom been explored as a framework for global health theory and practice.

## Current Applications of Complexity Theory in Global and Public Health

According to Pearce and Merletti [38], applying this emerging worldview to public health can lead to a more comprehensive analysis of public health challenges. For example, by asking questions beyond specific health phenomena from a biomedical perspective (e.g., malaria in a given community) and focusing on processes and relationships amongst system components in relation to health challenges (e.g., health systems, environmental context, international funding, historical aspects, sociocultural variability in relation to malaria – following the example above), CT’s application can reveal findings that can create more comprehensive solutions (i.e., emergence of new ways of understanding) at a local level. In this sense, CT seeks system-wide approaches and not *silver bullet* solutions. Similarly, but inversely, Frenk et al [14]. highlight the importance of attaining diverse global knowledge (e.g., of global health governance mechanisms) to achieve effective and equitable actions at a local level.

The very nature of complexity demands a transdisciplinary way of thinking and acting. Jogerst et al. [1] and Martens et al. [5] argue that cross-fertilization of disciplines plays a vital role in the face of current challenges due to the emerging and pluralistic contextual knowledge of global health phenomena. Simultaneously, many Indigenous scholars have argued that only through transdisciplinary approaches that incorporate many ways of knowing can we achieve our common goals [39].

Frenk et al. [14] have additionally suggested, albeit with other terminology, that applying the multiply ways of knowing included in CT will likely shift some actors’ roles in global health practice. This is especially important for the meaningful inclusion of communities in global health. Parkes et al. [40] suggest that integrating CT into health practice, in their case, the study of infectious disease, allowed for horizontal and vertical integration of stakeholders with a concomitant inclusion of perspectives and types of knowledge in infectious disease control. This approach, according to the authors, has the potential to create global health policy and action that is well-informed and contextualized to particular needs. Likewise, putting communities at the core of the health production process will allow comprehensive approaches representing core values of global health practice such as equity [5]. In this regard, CT has the potential to advance the global health equity agenda.

Another application of CT into the global health field has been in the analysis of global health governance. For example, Fidler [41] highlights that current theoretical models to understand health governance do not apply to global health, in part due to what he calls “open-source anarchy” (a fluid, interconnected system added to a lack of overarching governance structure to oversee global health governance processes). According to Haffeld [37] and Martens et al. [5], the understanding and framing of efficient global governance systems (one of the grand global health challenges in times of COVID-19) as complex adaptive social systems might also benefit from the application of the CT framework. Previous to this and in a 2009 report, the World Health Organization (WHO) stated that systems thinking (closely related to complex thought) “has huge and untapped potential, first in deciphering the complexity of an entire health system, and then in applying this understanding to design and evaluate interventions that improve health and health equity” [42 (pp.19)]. However, despite these calls to action, there is little evidence of these applications in practice, at least in the scientific literature.

Considering global health’s track record, one area of particular interest in global health practice that might benefit from the application of CT is that of the study of unintended consequences. Albrecht et al. [8], in explaining the role of CT, state “[A]t a minimum, trans-disciplinary understanding would suggest intervention avenues that would not make the problem worse, something that has occurred when culturally inappropriate or iatrogenic ‘solutions’ to health problems have been used”. Similarly, Kleinman [26] highlights the importance of providing tools for reflection in global health practice that can analyze the contextual conditions that lead to unintended consequences. Examples in the global health literature abound in this regard (see, for example, Biehl and Petryna [17]; Yamin [43]).

The tenets above, coupled with emergent findings from this approach (e.g., epidemiological complex modeling of infectious diseases like COVID-19), could redefine our understanding of global health as a complex discipline. It could also provide a framework for improved global health outcomes. The applications of CT into global health could also redefine and have profound implications for global health academia, including education, research, and practice, as has happened with other disciplines.

**Figure 2:**
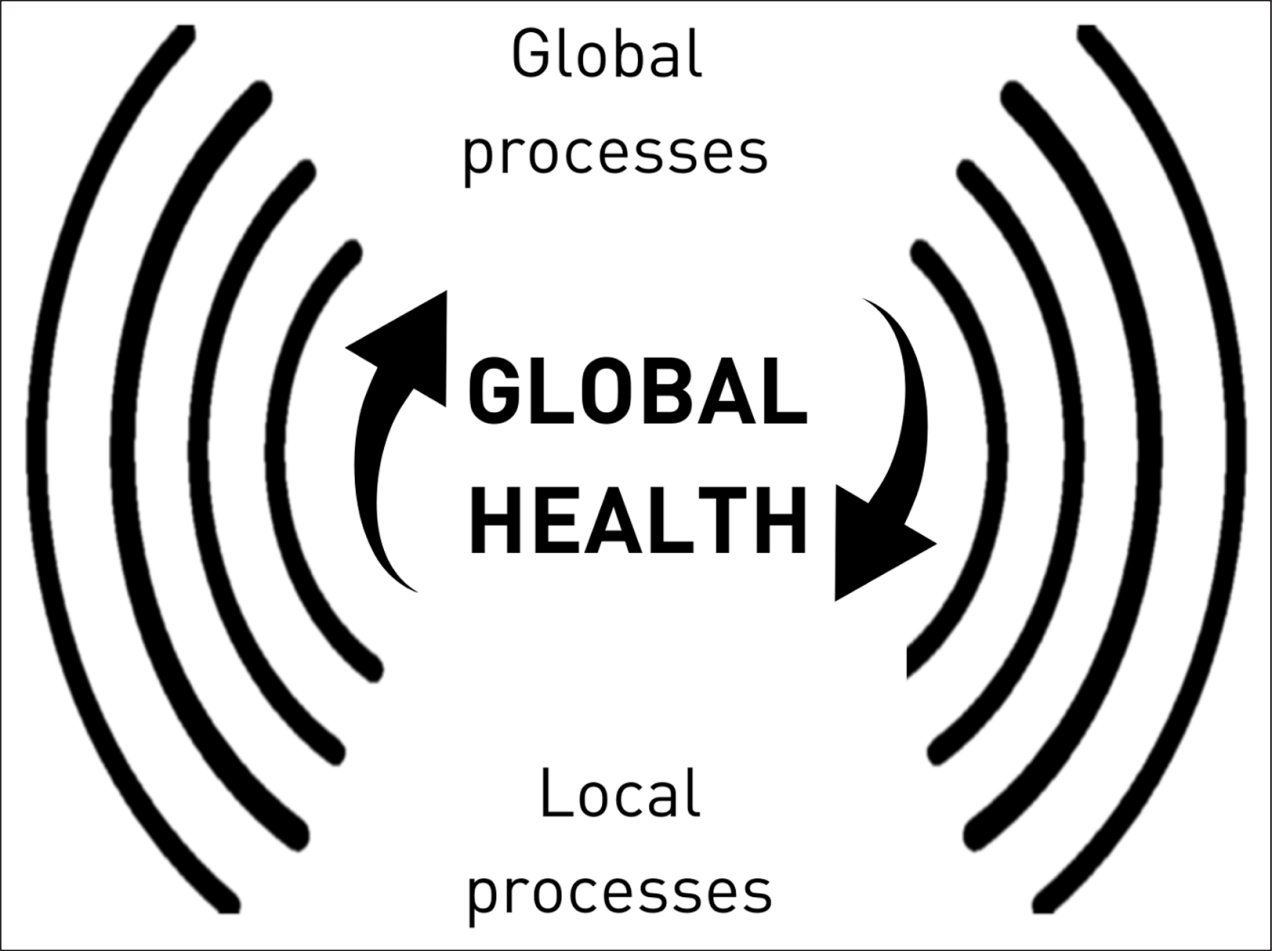
Global health as the study of the global and local processes that determine health and the interactions between these processes.

We undoubtedly live in a world with increasing global interdependence. This needs to be matched by the rise of capable practitioners that can understand the complex realities of current global health challenges like the COVID-19 syndemic. Therefore, and taking into consideration the gaps between global health theory and practice, in this viewpoint, I argue that the defining unifying theoretical framework of global health should be that of complexity theory. Stemming from this understanding, I propose that global health is, in essence, the study of the global and local processes that determine health and the interactions between these processes, as well as the emerging consequences of these interactions (Figure 2). This definition, although simple, is not to be confused with reductionist. On the contrary, it invites for the creation of a space that some have called “an ecology of knowledges” [44], and the integration of ongoing global health efforts.

## Future Directions

This article outlines the theoretical (or lack of) legacy that has dominated global health practice during the past decades. Aligned with the current dynamic and complex field of global health, the paper intends to explore the role of a new theoretical landscape using complexity theory to close the gaps of the aspirations put forth by the global health agenda. This approach is far from resolved, and questions such as the ones posed below still linger:

- How useful are CT concepts as explanatory models in quickly evolving global health phenomena?
- How efficient are CT concepts practical beyond explanatory models of global health phenomena? i.e., in seeking/creating solutions.
- How can CT be used to reduce the theory-to-practice gap in global health?
- When applied to global health, can CT increase the meaningful participation of historically marginalized communities and ways of knowing?
- To what extent and how could CT concepts be applied to global health education?

Although a nascent approach, the union between global health education/practice and complexity may have important epistemological and methodological implications for our understanding of the field. More importantly, it could improve human health by expanding future global health practitioners’ capacity and willingness to explore a wide variety of solutions to the pressing global health challenges of today and tomorrow.

## References

[1] Jogerst K, Callender B, Adams V, Evert J, Fields E, Hall T, et al. Identifying Interprofessional Global Health Competencies for 21st-Century Health Professionals. Annals of Global Health. 2015; 81(2): 239. DOI: 10.1016/j.aogh.2015.03.006 26088089

[2] Drain PK, Mock C, Toole D, Rosenwald A, Jehn M, Csordas T, Ferguson L, Waggett C, Obidoa C, Wasserheit JN. The emergence of undergraduate majors in global health: systematic review of programs and recommendations for future directions. The American journal of tropical medicine and hygiene. January 11, 2017; 96(1): 16. DOI: 10.4269/ajtmh.16-0687 28077739 PMC5239686

[3] A Affun-Adegbulu C, Adegbulu O. Decolonising global (public) health: from Western universalism to global pluriversalities. BMJ Global Health. August 1, 2020; 5(8): e002947. DOI: 10.1136/bmjgh-2020-002947 PMC7443258 32819916

[4] Marmot M, Friel S, Bell R, Houweling TA, Taylor S. Closing the gap in a generation: health equity through action on the social determinants of health. The Lancet. 2008; 372(9650): 1661–1669. DOI: 10.1016/S0140-6736(08)61690-6 18994664

[5] Martens P, Huynen M, Akin S, Hilderink H, Soskolne CL. Globalisation and human health: complexity, links and research gaps. IHDP Update. 2011; 1: 2–6.

[6] Whitmee S, Haines A, Beyrer C, Boltz F, Capon AG, de Souza Dias BF, Ezeh A, Frumkin H, Gong P, Head P, Horton R. Safeguarding human health in the Anthropocene epoch: report of The Rockefeller Foundation–Lancet Commission on planetary health. The Lancet. 2015; 386(10007): 1973–2028. DOI: 10.1016/S0140-6736(15)60901-1 26188744

[7] Watson MF, Bacigalupe G, Daneshpour M, Han WJ, Parra-Cardona R. COVID-19 interconnectedness: Health inequity, the climate crisis, and collective trauma. Family process. September 2020; 59(3): 832–46. DOI: 10.1111/famp.12572 32589267 PMC7361773

[8] Albrecht G, Freeman S, Higginbotham N. Culture, Medicine and Psychiatry. March 1998; 22(1): 55–92. DOI: 10.1023/A:1005328821675 9657059

[9] Bronfenbrenner U. The Ecology of Human Development?. Cambridge, MA: Harvard University Press; 1979.

[10] Frenk J, Gómez-Dantés O, Moon S. From sovereignty to solidarity: a renewed concept of global health for an era of complex interdependence. The Lancet. 2014; 383(9911): 94–7. DOI: 10.1016/S0140-6736(13)62561-1 24388312

[11] Myers S, Frumkin H. Planetary health: protecting nature to protect ourselves. Island Press; August 13 2020. DOI: 10.5822/978-1-61091-966-1

[12] Arya A, Allison J. Standardising medical education’s approach to global health: Are we moving forward? In: Arya AN, Evert, J (eds.), Global Health Experiential Education: From Theory to Practice. 2017; 217–222. New York: Routledge. DOI: 10.4324/9781315107844-25

[13] Abimbola S, Pai M. Will global health survive its decolonisation? The Lancet. 2020; 396(10263): 1627–1628. London, England. DOI: 10.1016/S0140-6736(20)32417-X 33220735

[14] Frenk J, Chen L, Bhutta ZA, Cohen J, Crisp N, Evans T, et al. Health professionals for a new century: transforming education to strengthen health systems in an interdependent world. The Lancet. 2010; 376(9756): 1923–58. DOI: 10.1016/S0140-6736(10)61854-5 21112623

[15] Richardson ET. Epidemic illusions: on the coloniality of global public health. MIT Press; December 22, 2020. DOI: 10.7551/mitpress/12550.001.0001

[16] Packard RM. A history of global health: interventions into the lives of other peoples. Baltimore; 2016.

[17] Biehl JG, Petryna A. When People Come First: Critical Studies in Global Health. Princeton University Press; 2013. DOI: 10.23943/princeton/9780691157382.001.0001

[18] Lalonde M. A new perspective on the health of Canadians. Ottawa: Government of Canada. Leavell H, Clark; 1974.

[19] World Health Organization. Declaration of Alma-Ata: International Conference on Primary Health Care, Alma-Ata. USSR. September 1978; 6–12.

[20] Bozorgmehr K. Rethinking the ‘global’ in global health: a dialectic approach. Globalization and Health. 2010; 6(1): 19. DOI: 10.1186/1744-8603-6-19 21029401 PMC2987787

[21] Holst J. Global Health–emergence, hegemonic trends and biomedical reductionism. Globalization and Health. December 2020; 16(1): 1–1. DOI: 10.1186/s12992-020-00573-4 32375801 PMC7201392

[22] Waggett CE, Jacobsen KH. Global health and public health majors and minors at 411 universities, 2019–2020. Annals of Global Health. 2020; 86(1). DOI: 10.5334/aogh.2837 PMC7304449 32587815

[23] Koplan JP, Bond TC, Merson MH, Reddy KS, Rodriguez MH, Sewankambo NK, et al. Towards a common definition of global health. The Lancet. 2009; 373(9679): 1993–5. DOI: 10.1016/S0140-6736(09)60332-9 PMC9905260 19493564

[24] Rowson M, Willott C, Hughes R, Maini A, Martin S, Miranda JJ, et al. Conceptualising global health: theoretical issues and their relevance for teaching. Globalization and Health. 2012; 8(1): 36. DOI: 10.1186/1744-8603-8-36 23148788 PMC3549856

[25] Farmer P. Reimagining Global Health: An Introduction. University of California Press; 2013.

[26] Kleinman A. Four social theories for global health. The Lancet. 2010; 375(9725): 1518–9. DOI: 10.1016/S0140-6736(10)60646-0 20440871

[27] Ablah E, Frenk J, Burke D, Spencer HC, Finnegan JR, Shortell S, et al. Improving Global Health Education: Development of a Global Health Competency Model. The American Journal of Tropical Medicine and Hygiene. 2014; 90(3): 560–5. DOI: 10.4269/ajtmh.13-0537 24445206 PMC3945704

[28] Rowthorn V, Olsen J. All Together Now: Developing a Team Skills Competency Domain for Global Health Education. Journal of Law, Medicine & Ethics. 2014; 42(4): 550–63. DOI: 10.1111/jlme.12175 25565620

[29] Arya AN, Evert J. (eds.) Global Health Experiential Education: from theory to practice. 1st ed. New York: Routledge; 2019.

[30] Nicolis G. and Prigogine I. Exploring complexity: an introduction. New York, NY: Freeman; 1998.

[31] Morin E. Restricted Complexity, General Complexity. Worldviews, Science and Us. 2007; 1–25. DOI: 10.1142/9789812707420_0002

[32] Lewin R. Complexity: life at the edge of chaos. Chicago University Press; 1999.

[33] Davidson AW, Ray MA, Turkel MC. Nursing, caring, and complexity science: for human-environment well-being. New York: Springer Pub.; 2011. DOI: 10.1891/9780826125880

[34] Miller JH. A crude look at the whole: the science of complex systems in business, life, and society. 1st ed. New York: Basic Books; 2016.

[35] Wilson T, Holt T, Greenhalgh T. Complexity science: Complexity and clinical care. BMJ. 2001; 323(7314): 685–8. DOI: 10.1136/bmj.323.7314.685 11566836 PMC1121241

[36] Plsek PE, Greenhalgh T. Complexity science: The challenge of complexity in health care. BMJ. 2001; 323(7313): 625–8. DOI: 10.1136/bmj.323.7313.625 11557716 PMC1121189

[37] Haffeld J. Facilitative governance: Transforming global health through complexity theory. Global Public Health. 2012; 7(5): 452–64. DOI: 10.1080/17441692.2011.649486 22248181

[38] Pearce N, Merletti F. Complexity, simplicity, and epidemiology. International Journal of Epidemiology. June 1, 2006; 35(3): 515–9. DOI: 10.1093/ije/dyi322 16415326

[39] Redvers N. The value of global indigenous knowledge in planetary health. Challenges. December 2018; 9(2): 30. DOI: 10.3390/challe9020030

[40] Parkes MW, Bienen L, Breilh J, Hsu L-N, Mcdonald M, Patz JA, et al. All Hands on Deck: Transdisciplinary Approaches to Emerging Infectious Disease. EcoHealth. 2005; 2(4): 258–72. DOI: 10.1007/s10393-005-8387-y

[41] Fidler D. Architecture Amidst Anarchy: Global Health’s Quest for Governance. Digital Repository Maurer Law. Published 2011. Accessed January 30, 2020. https://www.repository.law.indiana.edu/facpub/329/.

[42] De Savigny D, Adam T. Alliance For Health Policy And Systems Research, World Health Organization. Systems Thinking for Health Systems Strengthening. Alliance For Health Policy And Systems Research; 2009.

[43] Yamin AE. Power, suffering, and the struggle for dignity: Human rights frameworks for health and why they matter. University of Pennsylvania Press; December 4, 2015. DOI: 10.9783/9780812292190

[44] Sousa Santos, B. Beyond Abyssal Thinking: From Global Lines to Ecologies of Knowledges. Review (Fernand Braudel Center). 2007; 30(1): 45–89.

